# Supplementary material for: A Retina‐Inspired Optoelectronic Synapse Using Quantum Dots for Neuromorphic Photostimulation of Neurons
Source: Adv Sci (Weinh). 2024 Mar 6;11(18):2401753. doi: 10.1002/advs.202401753 (PMC11095222; doi:10.1002/advs.202401753)
Supplement: Supplementary file 1 — Supporting Information [file ADVS-11-2401753-s001.pdf]

ADVANCED SCIENCE
Open Access

Supporting Information

for *Adv. Sci.*, DOI 10.1002/advs.202401753

A Retina-Inspired Optoelectronic Synapse Using Quantum Dots for Neuromorphic Photostimulation of Neurons

*Ridvan Balamur, Guncem Ozgun Eren, Humeyra Nur Kaleli, Onuralp Karatum, Lokman Kaya, Murat Hasanreisoglu and Sedat Nizamoglu**

Supplementary Materials for

A Retina-inspired Optoelectronic Synapse Using Quantum Dots for Neuromorphic Photostimulation of Neurons

Ridvan Balamur[1], Guncem Ozgun Eren[2], Humeyra Nur Kaleli[3], Onuralp Karatum[1], Lokman Kaya[1], Murat Hasanreisoglu[3], Sedat Nizamoglu[1,*]

[1]*Department of Electrical and Electronics Engineering, Koc University, Istanbul, Turkey*

[2]*Department of Biomedical Science and Engineering, Koc University, Istanbul, Turkey*

[3]*Research Center for Translational Medicine, Koc University, Istanbul, Turkey*

*snizamoglu@ku.edu.tr

This PDF file includes:

Supplementary Text

Tables S1 and S2

Figs. S1 to S11

References (1)

Table S1. Zinc and sulfur precursor solutions used for ZnS shelling.

	Concentration (M)	InP/0.5ZnS	InP/1ZnS
**$ZnSt_2$-ODE**	0.1	600 µL	1050 µL
**S-TOP**	0.1	600 µL	1050 µL

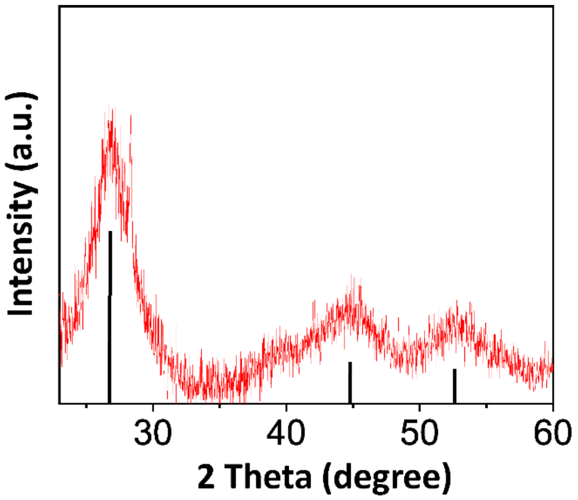

Figure S1. XRD pattern of the InP/ZnS core/shell QDs. The X-ray diffraction (XRD) pattern reveals the crystal planes of the (111), (220), and (222) of the QDs. (InP JCPDS No. 32-0452 and ZnS JCPDS No. 80-0020)

The XRD peak positions of the resulting InP/ZnS core/shell QDs at 2θ values of 26.93°, 45.11° correspond to cubic (zinc-blende) crystal structure of InP (JCPDS No. 32-0452). Moreover, we observed no additional peaks related to separate ZnS formation. Therefore, we can conclude that ZnS formation occurs on InP core structure as shell material with cubic crystal structure (JCPDS No. 80-0020) [1-3]. In this regard, the thickness of the ZnS shell growth was calculated based on previously reported studies [4, 5] as follows:

The lattice constant of zinc-blende ZnS: a=0.540 nm

The average thickness of one monolayer of ZnS with zinc-blende crystal structure (d) can be calculated using Equation 1:

$$d = \frac{\sqrt{3}}{3} \times a \quad (1)$$

According to Eq. 1 the average shell thickness of one monolayer is 0.313 nm. Considering the TEM analysis, after one ZnS shell growth, the mean size of QDs increased from 2.49 nm to 2.81 nm and the shell thickness equals to 0.16 nm shell thickness corresponding to 0.51 monolayers (ML).

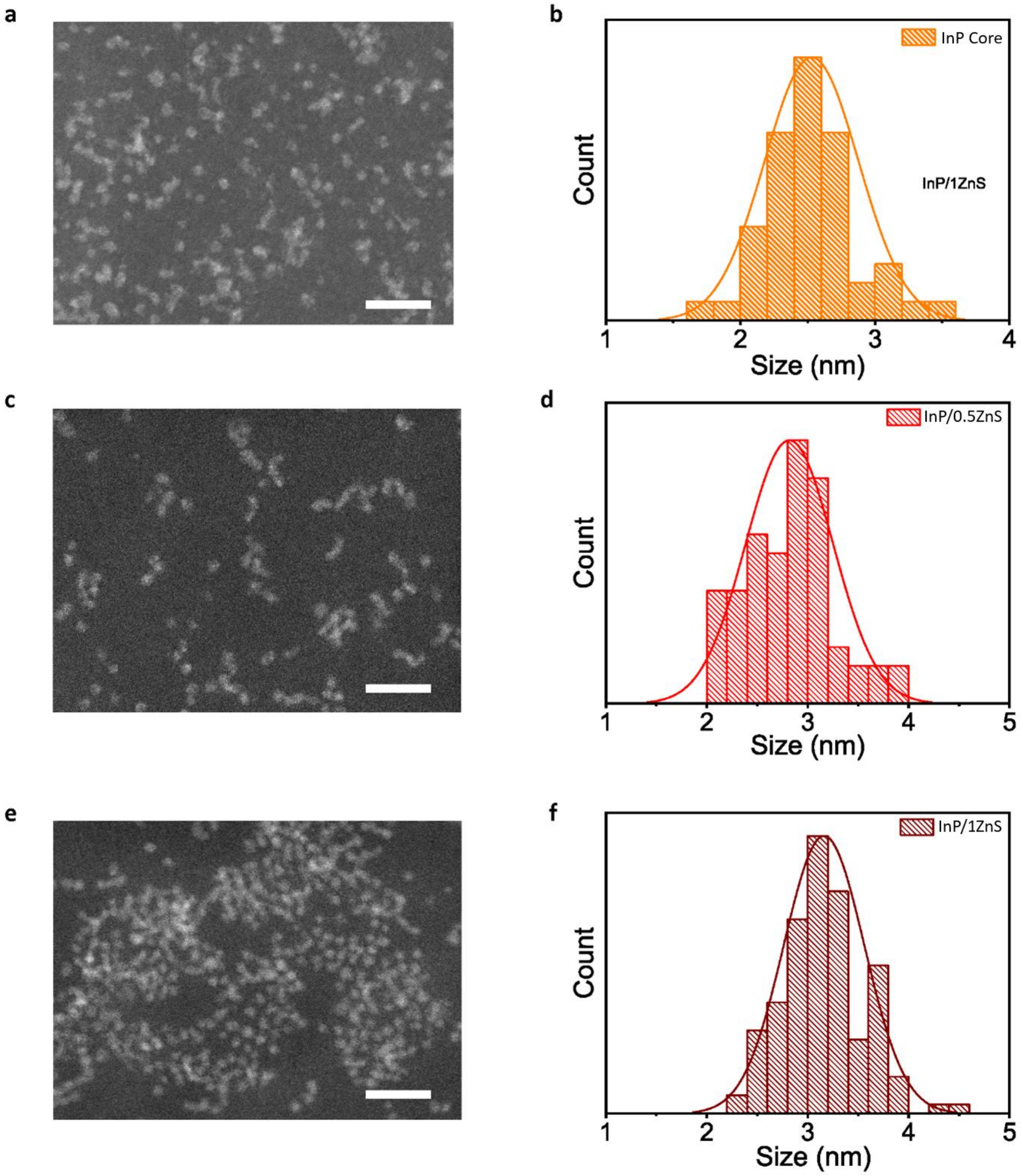

Figure S2. (a) TEM image of InP core QDs. **(b)** Size distribution of InP core QDs where the mean size is 2.49 nm. **(c)** TEM image of InP/0.5ZnS core/shell QDs. **(d)** Size distribution of InP/0.5ZnS core/shell QDs where the mean size is 2.81 nm. **(e)** TEM image of InP/1ZnS core/shell QDs. **(f)** Size distribution of InP/3ZnS core/shell QDs where the mean size is 3.15 nm. 200 QDs are counted for each size distribution plot. Scale bars of TEM images are 20 nm.

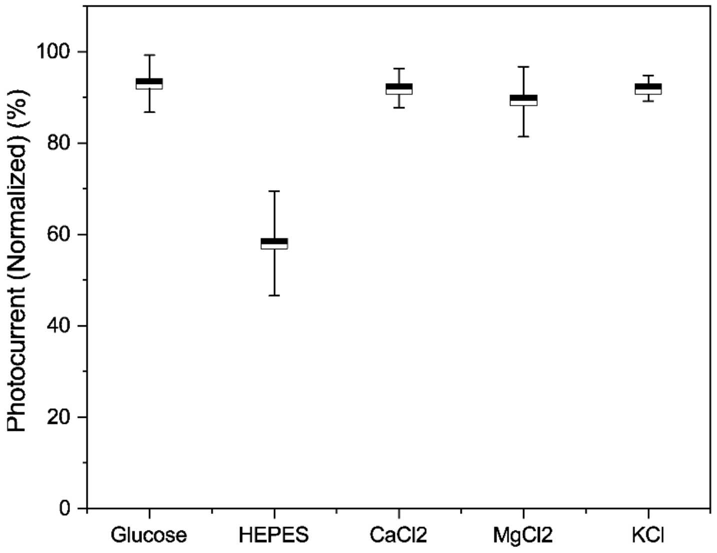

Figure S3. Photocurrents while each ingredient is decreased to their half (means ± SD, n=5).

Table S2. Signal-to-Noise ratio of electrochemistry and electrophysiology

SNR of electrochemistry	SNR of electrophysiology setup	
	Subthreshold condition	Suprathreshold condition
40.5 dB	17.5 dB	30.9 dB

Table S2 shows the signal-to-noise ratio (SNR) for electrochemistry and electrophysiology characterizations. An SNR of 40.5 dB is observed in the electrochemistry with a 5 ms light pulse, indicating a robust signal quality. Meanwhile, the electrophysiology can be analyzed under two conditions, subthreshold and suprathreshold conditions, where the occurrence of AP will vary the SNR values. With an action potential, the SNR is 30.9 dB, highlighting signal clarity during dynamic physiological events. On the other hand, the SNR with 5 ms light pulse is recorded as 17.5 dB at rest.

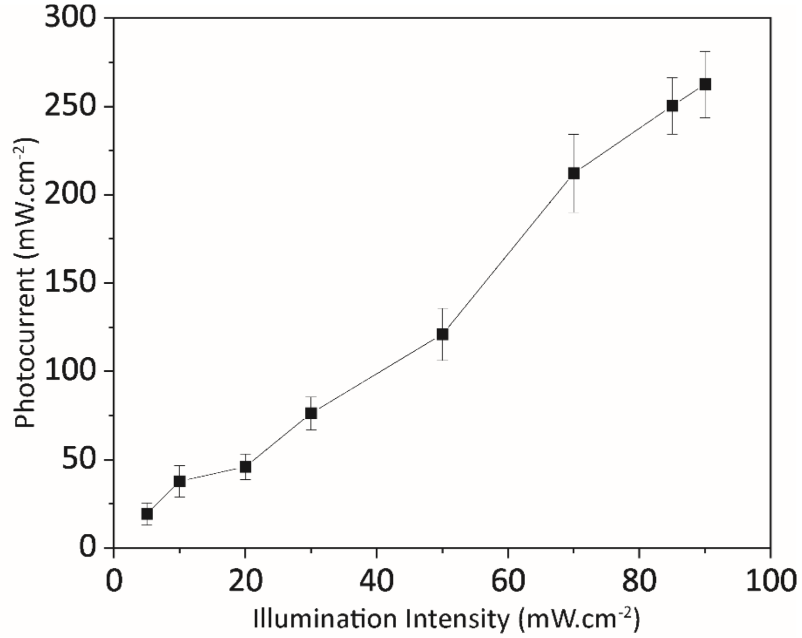

Figure S4. Photocurrent measurements in the aCSF electrolyte under 20 ms illumination under different light intensities (means ± SD, n=5).

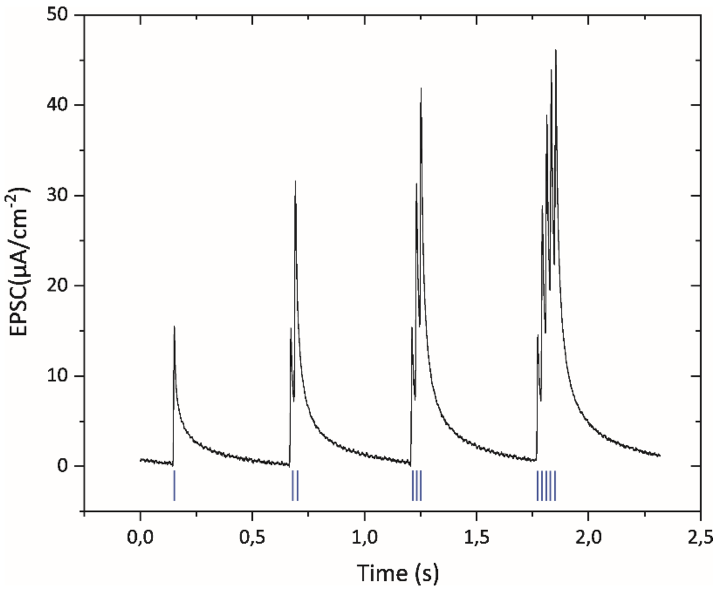

Figure S5. Spike-number-depended-plasticity (SNDP) for 1,2,3, and 5 pulses.

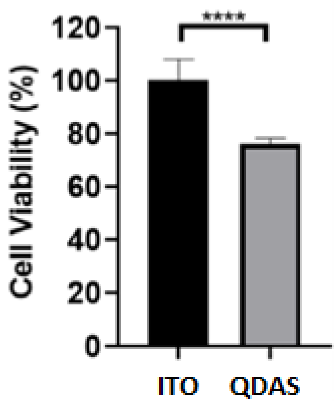

Figure S6. Biocompatibility analysis of QDAP compared to ITO control. CTG cell viability result of primary hippocampal neurons cultured on QDAPs and ITO substrates (mean ± SD for n = 4). An unpaired, two-tailed t-test was used for statistical analysis, and ****p < 0.0001 was evaluated as statistically significant.

~80% biocompatibility indicates that it is generally well-tolerated by cells and has a relatively low level of adverse effects. Such biocompatibility levels are appropriate to grow primary neurons on the device for photostimulation [6].

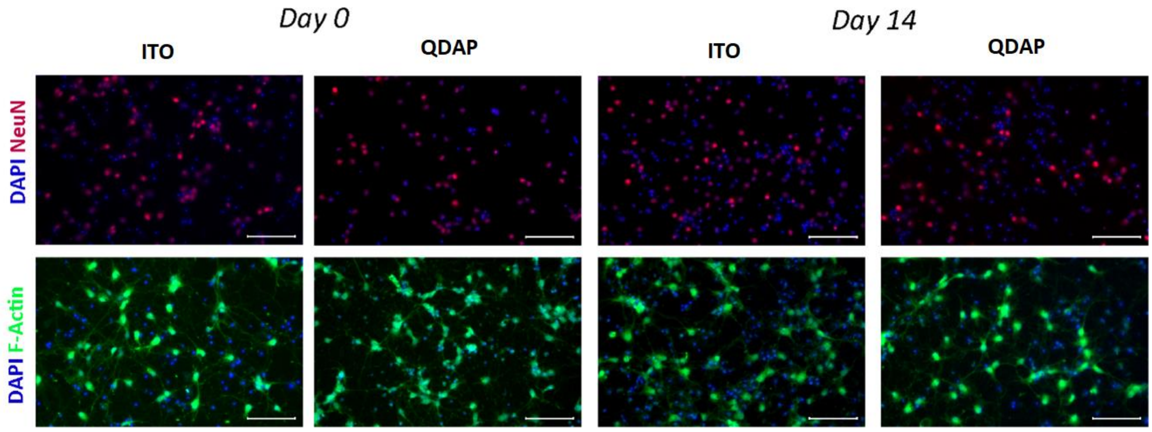

Figure S7. Immunofluorescence images of primary hippocampal neurons cultured on QDAP and ITO substrates on days 0 and 14 of culturing to observe morphology and viability of primary hippocampal neurons. Cells were co-stained with DAPI (blue) to show the nucleus, Anti-NeuN (red) to show the neuronal nucleus, and Anti-f-Actin (green) to indicate cell structure (scale bar: 100 µm).

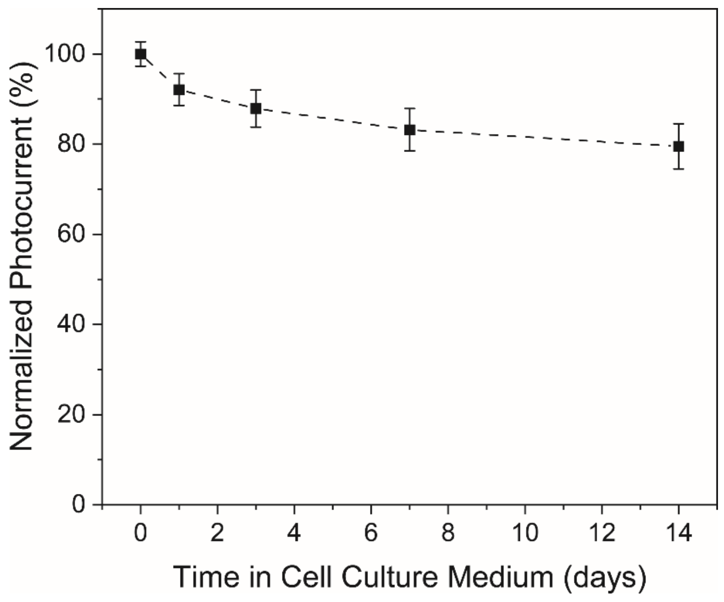

Figure S8. Normalized Photocurrent peak up to 14 days of cell culture.

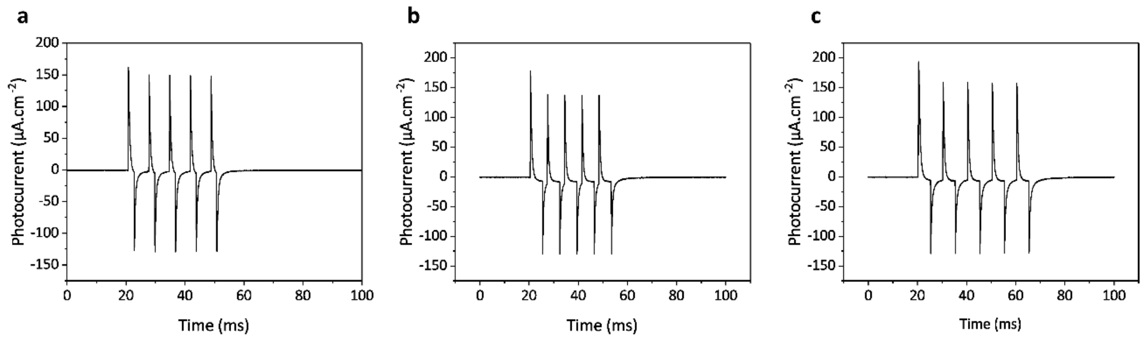

Figure S9. Capacitive photocurrent response of ITO/ZnO/P3HT device triggered with different illuminations, (left) illuminated with 2 ms on time and 5 ms off time, (middle) illuminated with 5 ms on time and 5 ms off time, (right) illuminated with 5 ms on time and 2 ms off time.

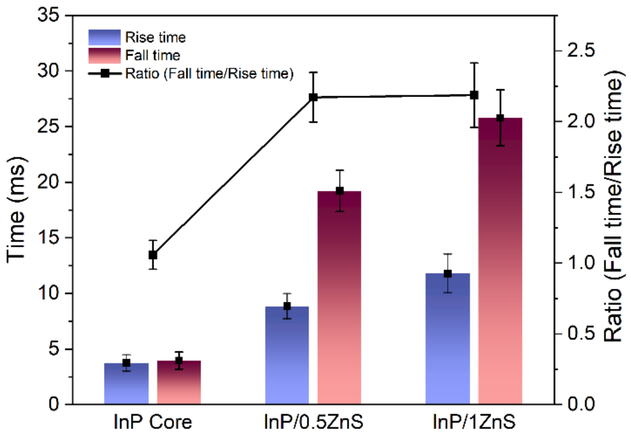

Figure S10. Rise time, fall time, fall time/rise time analysis of the QDAP devices with different QD nanostructures.

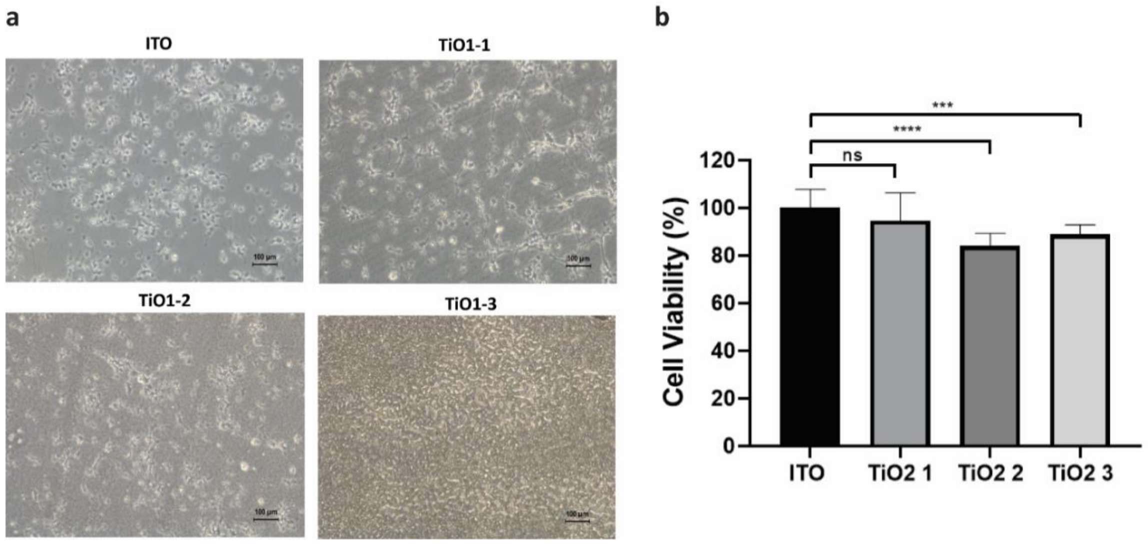

Figure S11. Biocompatibility analysis of ITO/TiO_2 thin film compared to ITO control. **a** Images of primary hippocampal neurons cultured on ITO/TiO_2 thin films and ITO substrate to observe morphology and viability of primary hippocampal neurons (scale bar: 100 µm). **b** CTG cell viability result of primary hippocampal neurons cultured on ITO/TiO_2 and ITO substrates (mean ± SD for n = 4). An unpaired, two-tailed t-test was used for statistical analysis, and ****$p < 0.0001$ was evaluated as statistically significant. TiO2-1, TiO2-2, and TiO2-3 corresponds to 50, 100 and 200 nm TiO2 thickness, respectively.

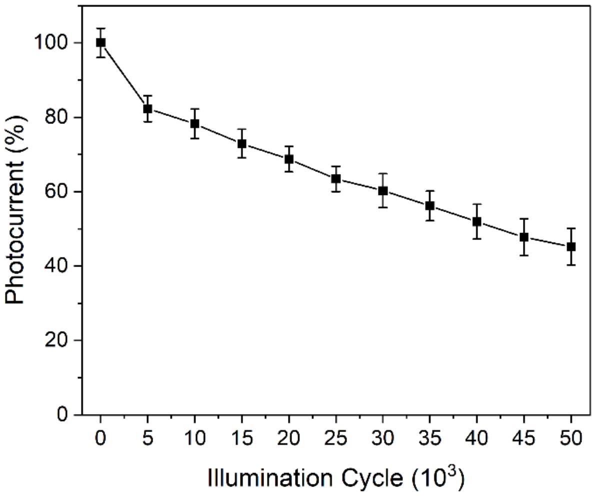

Figure S12. Lifetime of the QDAS (mean ± SEM, n = 5) under the stimulus frequency of 5 Hz, pulse-width of 2 ms, optical power density of 10 mW.cm^{-2}.

References:

1. Zhang, W., et al., *High Quantum Yield Blue InP/ZnS/ZnS Quantum Dots Based on Bromine Passivation for Efficient Blue Light-Emitting Diodes.* Advanced Optical Materials, 2022. **10**(15): p. 2200685.
2. Li, L. and P. Reiss, *One-pot synthesis of highly luminescent InP/ZnS nanocrystals without precursor injection.* Journal of the American Chemical Society, 2008. **130**(35): p. 11588-11589.
3. Suh, Y.H., et al., *Engineering core size of InP quantum dot with incipient ZnS for blue emission.* Advanced Optical Materials, 2022. **10**(7): p. 2102372.
4. Taniguchi, S. and M. Green, *The synthesis of CdTe/ZnS core/shell quantum dots using molecular single-source precursors.* Journal of Materials Chemistry C, 2015. **3**(32): p. 8425-8433.
5. Chen, D., et al., *Bright and stable purple/blue emitting CdS/ZnS core/shell nanocrystals grown by thermal cycling using a single-source precursor.* Chemistry of Materials, 2010. **22**(4): p. 1437-1444.
6. Schmidt, T., et al., *Light stimulation of neurons on organic photocapacitors induces action potentials with millisecond precision.* Advanced Materials Technologies, 2022. **7**(9): p. 2101159.
